# Orthodontic Traction of Impacted Canine Using Cantilever

**DOI:** 10.1155/2016/4386464

**Published:** 2016-10-09

**Authors:** Cláudia Nakandakari, João Roberto Gonçalves, Daniel Serra Cassano, Taísa Boamorte Raveli, Jonas Bianchi, Dirceu Barnabé Raveli

**Affiliations:** ^1^Araraquara School of Dentistry, Universidade Estadual Paulista (UNESP), Rua Humaitá 1680, 14801-90 Araraquara, SP, Brazil; ^2^Private Practice, Avenida Doutor Gastão Vidigal 295, 14802-408 Araraquara, SP, Brazil

## Abstract

The impaction of the maxillary canines causes relevant aesthetic and functional problems. The multidisciplinary approach to the proper planning and execution of orthodontic traction of the element in question is essential. Many strategies are cited in the literature; among them is the good biomechanical control in order to avoid possible side effects. The aim of this paper is to present a case report in which a superior canine impacted by palatine was pulled out with the aid of the cantilever on the Segmented Arch Technique (SAT) concept. A 14.7-year-old female patient appeared at clinic complaining about the absence of the upper right permanent canine. The proposed treatment prioritized the traction of the upper right canine without changing the occlusion and aesthetics. For this, it only installed the upper fixed appliance (Roth with slot 0.018), opting for SAT in order to minimize unwanted side effects. The use of cantilever to the traction of the upper right canine has enabled an efficient and predictable outcome, because it is of statically determined mechanics.

## 1. Introduction

Facial harmony is directly associated with the presence of canines in the dental arch, which are important for stable occlusion [1]. However, tooth impaction can be one of the factors responsible for the aesthetic and functional imbalance. In particular, the incidence of impaction of the upper canines ranges from 1% to 3% [2]. Some etiological factors may justify the retention and/or impaction of the canine, as the long path that runs to its full eruption associated with the fact that is the last tooth to erupt in chronological order, besides genetic factors, atresic arcade, trauma, and consequences of systemic diseases [1, 3].

The impaction of maxillary canines occurs most frequently by palatine (85% against 15% in the buccal region). For a correct diagnosis and development of the treatment plan, it is essential to define the tooth location. Thus, it is essential to perform a detailed clinical examination, associated with radiographic and/or computed tomography. Clinical signs observed in cases of impaction are prolonged retention of deciduous canine, delayed eruption of the permanent canine and, depending on the position of the canine included, absence of vestibular bulging, presence of palatal bulging, and distal slope of the maxillary lateral incisor crown and may present, or not, midline deviation [4].

The orthodontic treatment should be started as soon as possible to avoid secondary problems [5]. One of the most suitable procedures is orthodontic traction, and its success is directly linked to the management of side effects. Therefore, biomechanical knowledge is required to choose an ideal system of forces for each intended movement [6].

The Segmented Arch Technique (SAT) consists in the dental arch segmentation for the consolidation of teeth in active units and a passive unit (anchor), being designed by Charles Burstone in 1962. This technique can be applied to the cantilever, which is a device used for dental traction, being made from titanium and molybdenum alloy (TMA) wire. A lever arm inserted into a tube (anchorage unit) is connected to an active unit (impacted canine), tieding to a free end. To increase system flexibility, helicoids can be made close to the fixed end. The anchor block allows obtaining maximum stability of the posterior segment through the transpalatal arch (TPA) use, which reduces undesirable effects [7, 8].

The aim of this paper is to present a case in which the upper canine impacted by palatine was pulled out with cantilever aid in SAT concept in order to minimize the side effects and increase the effectiveness of treatment.

## 2. Case Report

**Figure 1:**
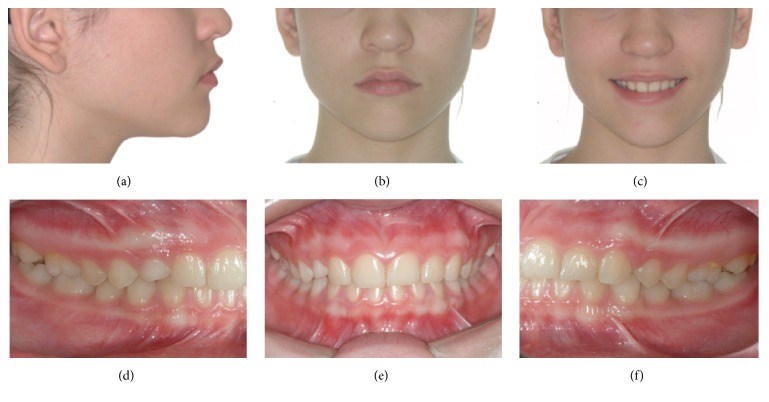
Initial documentation: profile photo (a); frontal photo (b); frontal photo with smile (c); intraoral photos: right, (d) frontal (e), and left side (f).

**Figure 2:**
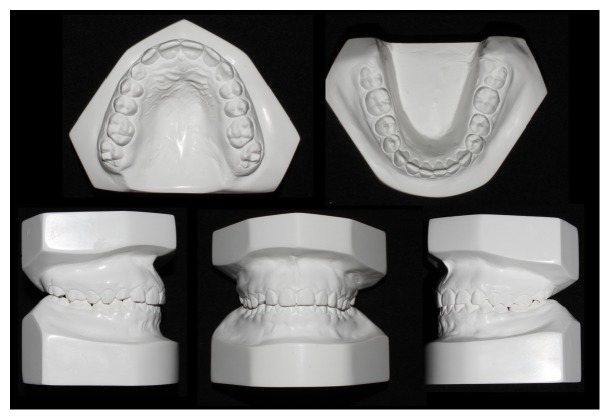
Initial documentation: study models showing a suitable arch shape and a good occlusal relationship.

**Figure 3:**
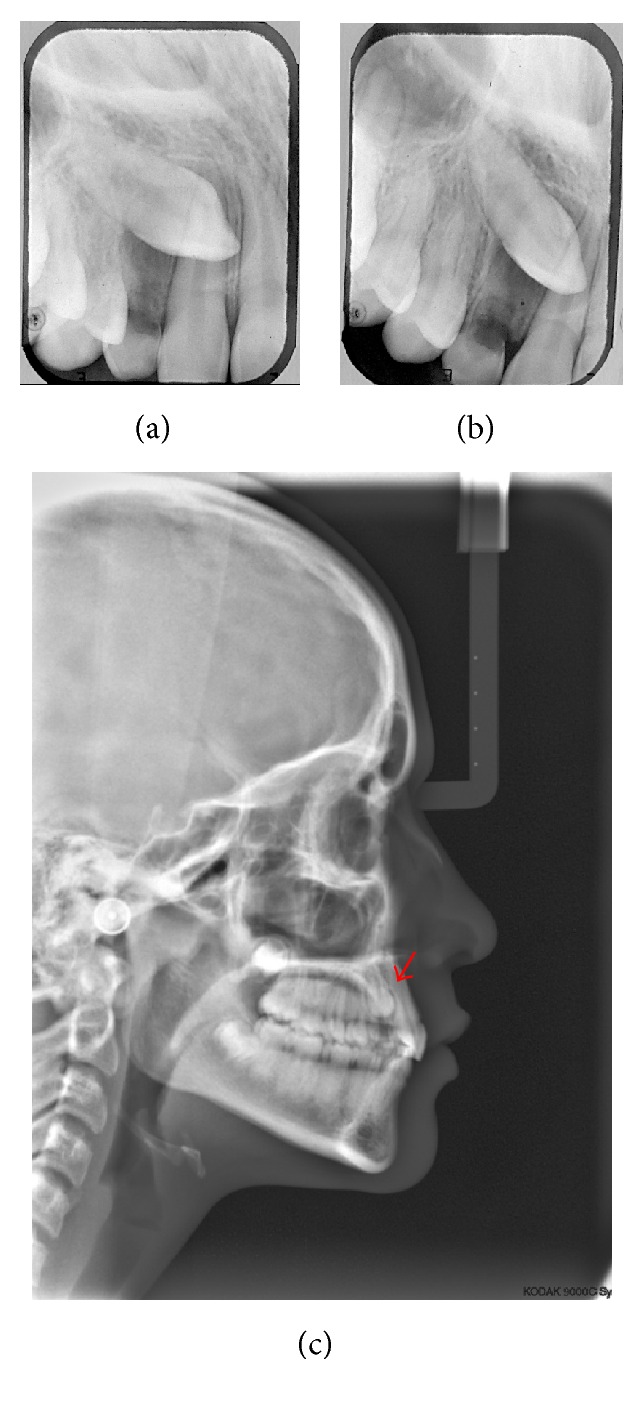
Initial documentation: exams imaging for diagnosis of impacted canine. (a) and (b) are periapical radiographs with Clark technique (note that in (b) the tooth moved distally, indicating its position by palatal); red arrow indicates the impacted tooth (c).

A 14.7-year-old female patient appeared at clinic complaining about the absence of the upper right permanent canine. Clinical examination showed symmetrical and proportional face, straight and harmonious profile, passive lip closure, suitable smile line (Figures 1(a)–1(c)), Angle Class I occlusion, matching media lines, normal overjet and overbite, mixed dentition with presence of the element 53, and the absence of its successor 13 (Figures 1(d)–1(f)). The models confirm the occlusion in Angle Class l with parabolic arches and suitable overjet and overbite (Figure 2). Through Clark technique, the palatal position of the canine was observed (Figure 3).

The proposed treatment prioritized the traction of the upper right canine without changing the occlusion and aesthetics. For this, it only installed the upper fixed appliance (Roth with slot 0.018), opting for SAT in order to minimize unwanted side effects. After arch alignment and leveling, the anchorage unit was composed of a stainless steel arch (0.017′′ × 0.025′′) passing passively on all upper teeth, except for the canine.

**Figure 4:**
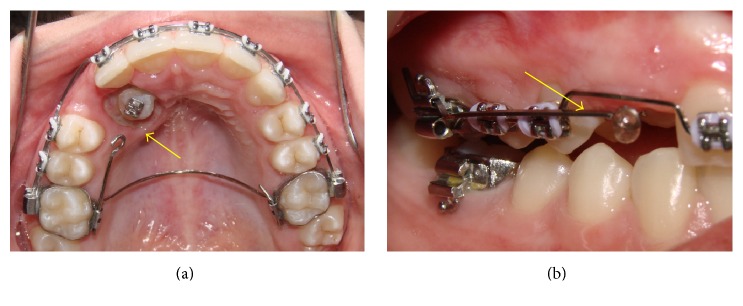
Intraoral photographs. Access to the impacted tooth through open surgery (a). The arrow points to the cantilever made from the transpalatal arch extension; cantilever in TMA coupled to the auxiliary band tube (b).

A modified Transpalatal Arch (TPA) with a stretched arm at traction side was used. At the end of this arm, the extrusion force directed to impacted tooth was applied (Figure 4). After mounting of the anchoring system, the patient underwent surgery for bonding orthodontic button. Firstly, the closed technique was held to access the tooth. However, the accessory became unglued during traction and an open tunnel surgery was performed to guide the tooth trajectory with an orthodontic bracket placed on the exposed crown (Figure 4(a)) [9, 10]. A second cantilever, made with TMA wire (0.017′′ × 0.025′′), was coupled by buccal to maximize the biomechanics effects (Figure 4(b)).

**Figure 5:**
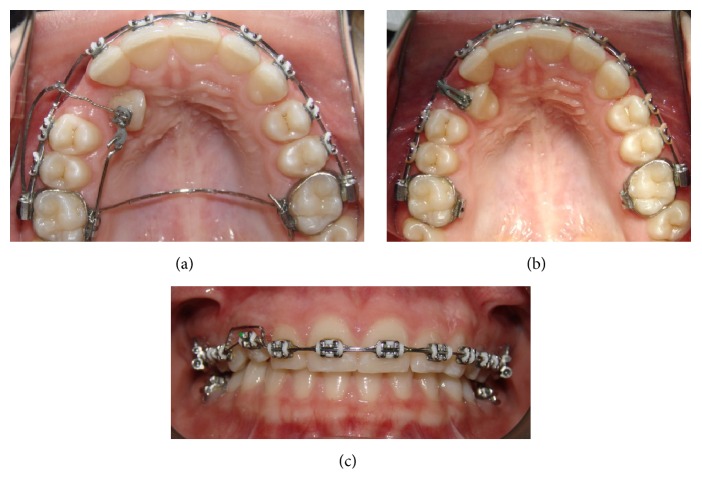
Intraoral photographs. Mechanism of action of the two cantilevers acting together (a). Removal of the transpalatal arch and cantilever coupled to the tube (b). Bypass in the region of upper right canine and round wire assisting in the mechanical forces (c).

**Figure 6:**
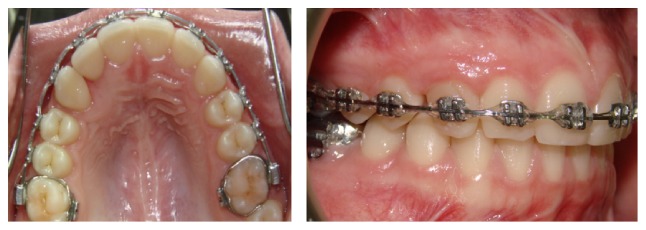
Intraoral photos after 2 months of using the NiTi wire (0.016).

After traction, controlled with the aid of cantilevers (Figures 5(a) and 5(b)), the final positioning of impacted tooth was performed using a rectangular rigid steel wire (0.017′′ × 0.025′′) containing a bypass for canine region. Thus, a round wire of NiTi (0.016) was added to the system of forces, causing minimal side effects and helping the canine to achieve the optimum position (Figure 5(c)). In Figure 6, the result after 2 months of NiTi wire use is observed.

**Figure 7:**
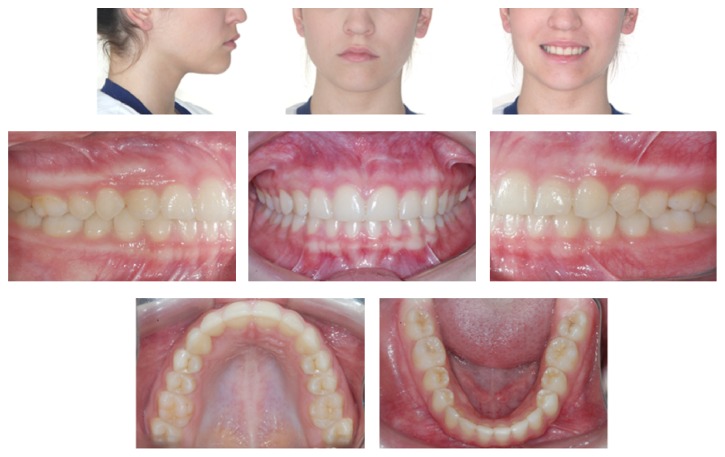
Photos immediately after removal of the appliance.

**Figure 8:**
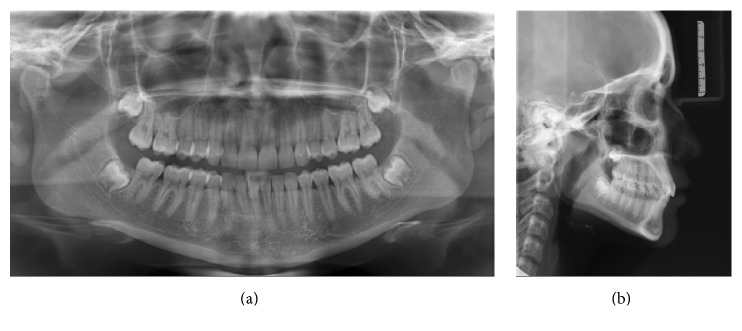
Final radiographs. In panoramic radiography, good positioning of all erupted teeth and parallelism of roots are observed (a); in teleradiography it is noted good maxillomandibular relationship as well as between the upper and lower incisors (b).

Figure 7 shows the end result of orthodontic treatment. The mechanics have remained very punctual, without side effects, preserving the initial characteristics of the patient. In the radiographic evaluation (Figure 8), the normality of tooth positions is noted, in addition to satisfactory standard facial.

## 3. Discussion

The permanent maxillary canines have significant incidence of impaction, being the most affected teeth after the third molars. The impaction of the canine is more common in the maxilla, palatally with unilateral trend. It is three times more common in females and occurs even when there is the presence of enough space to align the arch [11].

In this case, ectopic position and exaggerated horizontal inclination of canine resulted in its impaction. The diagnosis was confirmed based on radiographic evaluation. No history of trauma or family report.

Based on the initial analysis of the impacted tooth position, treatment prioritized three orthodontic movements: extrusion, verticalization, and vestibularization. Initially, the cantilever was activated to verticalize and expose the tooth crown in the oral environment, by applying a force with a component mainly extrusive. In the vestibular movement, the canine was displaced towards the arc and at extrusion, the cantilever has leveled the canine [12]. Thus, the function has been established, besides position and aesthetic tooth in the occlusion; at the same time, the integrity of the periodontium and surrounding structures was maintained [13].

In this clinical case, the SAT has enabled applying biomechanical principles to minimize the side effects generated by orthodontic appliances, regardless of patient cooperation. SAT is particularly suitable for working with statically determined force systems, mild and constant, avoiding unnecessary and unpredictable movements [7, 12, 14]. Although it requires more clinical time and the segmented handles promote certain discomfort to the patient, the SAT is an important tool for the orthodontist when continuous arch techniques are limited to control side effects and the desired result.

## 4. Conclusion

The use of Segmented Arch Technique to traction of the upper right canine has enabled an efficient and predictable outcome, minimizing side effects in orthodontic arch.
